# Decoding the Clinical Significance of Immunoglobulin G4 in Rheumatoid Arthritis

**DOI:** 10.3390/jcm12144716

**Published:** 2023-07-17

**Authors:** Li Fen Tan, Rajalingham Sakthiswary, Uma Rajeswaran Veshaaliini, Syahrul Sazliyana Shaharir, Asrul Abdul Wahab, Suraya Aziz, Rosnah Sutan

**Affiliations:** 1 Department of Medicine, Faculty of Medicine, Universiti Kebangsaan Malaysia, Hospital Canselor Tuanku Muhriz, Kuala Lumpur 56000, Malaysia; p100916@siswa.ukm.edu.my (L.F.T.); p117636@siswa.ukm.edu.my (U.R.V.); syahrul.sazliyana.shaharir@ppukm.ukm.edu.my (S.S.S.); 2 Department of Immunology, Faculty of Medicine, Universiti Kebangsaan Malaysia, Hospital Canselor Tuanku Muhriz, Kuala Lumpur 56000, Malaysia; saw@ppukm.ukm.edu.my; 3 Department of Radiology, Faculty of Medicine, Universiti Kebangsaan Malaysia, Hospital Canselor Tuanku Muhriz, Kuala Lumpur 56000, Malaysia; drsuraya@ppukm.ukm.edu.my; 4 Department of Public Health Medicine, Faculty of Medicine, Universiti Kebangsaan Malaysia, Hospital Canselor Tuanku Muhriz, Kuala Lumpur 56000, Malaysia; rosnah.sutan@ukm.edu.my

**Keywords:** immunoglobulin G4, rheumatoid arthritis, disease activity, joint damage, functional disability, treatment response

## Abstract

Immunoglobulin (Ig) G4 accounts for 4–6% of the total IgG in a healthy human. Several evidence-based studies have suggested that the level of IgG4 is significantly elevated in autoimmune diseases, including rheumatoid arthritis (RA). The clinical significance of IgG4 in RA with regard to disease activity, severity, and treatment response remains elusive. We consecutively recruited 174 patients with RA from our rheumatology clinic. All subjects were assessed for their disease activity based on DAS28, radiographic joint damage based on the Modified Sharp Score (MSS), the functional capacity based on the Health Assessment Questionnaire –Disability Index (HAQ-DI), and treatment responsiveness using the European League Against Rheumatism (EULAR) response criteria. The serum IgG4 of the recruited subjects was measured via the ELISA test. The mean serum IgG4 level was 60.23 ± 30.08 mg/dL. We found that serum IgG4 had significant positive correlations with disease activity (r = 0.406; *p* < 0.001), ESR (r = 0.155; *p* = 0.041), CRP (r = 0.269; *p* < 0.001), joint damage (r = 0.195; *p* = 0.012) and functional disability (r = 0.909; *p* < 0.001). Subjects with elevated IgG4 (IgG4 > 86 mg/dL) had significantly higher ESR, CRP, HAQ-DI, and DAS 28 and a poorer treatment response compared to the group with non-elevated IgG4. After multivariate analysis, only HAQ-DI (OR = 4.229, 95% CI 1.302, 15.751, *p* = 0.018) and DAS28 (OR = 3.743, 95% CI 1.062, 13.193, *p* = 0.040) remained significantly associated with elevated serum IgG4. The preliminary findings of this study could suggest serum IgG4 to be a potential biomarker of disease activity and functional disability in RA.

## 1. Introduction

Rheumatoid Arthritis (RA) is the most common form of symmetrical inflammatory arthritis. The peripheral small (hands, feet, and wrists) and large (knees, elbows, ankles, and shoulders) joints may be involved in RA. It affects millions of people in this country, especially women. Adults of all age groups can be affected by this disease [1]. RA is a systemic autoimmune disease with complex pathogenesis. Understanding the mechanisms involved is pivotal to the successful development of effective therapies. If left untreated, this disease can lead to severe joint damage causing physical disability and immobility. The socioeconomic consequences of this disease on society should not be underestimated.

Immunoglobulins (Ig) are structurally unique proteins that consist of two pairs of heavy and light chains. It plays a pivotal role in autoimmune diseases such as RA, systemic lupus erythematosus (SLE), and myasthenia gravis. These proteins are subclassed into IgA, IgD, IgG, and IgM. IgG is the major immunoglobulin class that makes up about 80% of the total human serum Ig [2]. IgG can be subdivided into four subclasses, i.e., IgG1 (60–70%), IgG2 (15–20%), IgG3 (5–10%), IgG4 (4–6%) [3]. Over the past few decades, much attention has been drawn to IgG4 since the recognition of IgG4-related disease (IgG4-RD) in 2011 [4]. IgG4 is a rather unique form of IgG. Unlike other IgG subclasses, IgG4 antibodies do not bind to the complement or stimulate the classical complement pathway and are poor Fc receptor activators. These properties of IgG4 can attenuate inflammation and suppress hypersensitivity through the inhibition of IgE [5]. IgG4-RD is a multiorgan for inflammation and fibrosis with histologically marked inflammatory infiltrates of IgG4-positive plasma cells and elevated serum IgG4 [5].

The IgG4-autoimmune disease (IgG4-AIDs) is characterized by autoantibody responses predominantly of the IgG4 subclass against a known antigen. These disorders can affect many organ systems, including the kidneys, nervous system, haematopoietic system, and skin. As IgG4 is predominantly anti-inflammatory, the pathogenic effects are speculated to be from the blocking of essential protein–protein interactions from the target antigen. The pathological consequences of IgG4 autoantibodies may differ from one disease to another [6].

Recent studies have shown how serum IgG4 was significantly elevated in the RA population. However, the precise role of IgG4 in RA and the rest of the autoimmune diseases remains to be elucidated [7]. In RA, interleukin (IL)-6 contributes to the production of IgG4-specific anti-CCP autoantibodies, most probably through the upregulation of IL-21 production by CD4 T cells [8]. The CH2 domain of IgG4 has the capacity to bind with autoantigens and rheumatoid factors (RFs) in RA. The immune complex containing RF-IgG4-autoantigens was shown to be proinflammatory in nature [9].

To date, there has been a lack of data on the correlation between serum IgG4 levels and the various clinical and biochemical aspects of RA, such as disease activity, joint damage, the responsiveness to diseases modifying anti-rheumatic drugs (DMARDs), and functional capacity. This prompted us to conduct a study to fill the aforementioned knowledge gap. We reckon that IgG4 is a potential therapeutic target in RA. Hence, the purpose of this study was to elucidate the clinical significance of IgG4 in RA with regard to disease activity, joint damage, functional disability, and treatment response.

## 2. Materials and Methods

### 2.1. Study Design 

This cross-sectional study was conducted in Hospital Canselor Tuanku Muhriz (HCTM), the National University of Malaysia, from June 2022 to April 2023, with the approval of the Research Ethics Committee of HCTM. Funding was obtained from the Fundamental Research Grant Scheme (FRGS/1/2021/SKK08/UKM/02/1) by the Ministry of Higher Education of Malaysia.

### 2.2. Study Population

Patients were recruited from the rheumatology clinic of HCTM. The inclusion criterion was patients with a confirmed diagnosis of RA based on the American College of Rheumatology 2010 classification criteria of RA [10]. We excluded patients with (a.) overlap syndrome alongside other connective tissue diseases such as Systemic Lupus Erythematosus, Systemic Sclerosis, and Polymyositis; (b.) pregnant RA patients whose radiographic assessment was contraindicated; (c.) patients with concomitant malignancy; and (d.) patients with acute infections.

We used convenient sampling; a non-probability sampling method was used whereby patients were approached during their scheduled clinic visits. All patients were counseled, and informed consent was obtained before recruitment into this study. 

### 2.3. Data Collection 

Demographic data such as age, gender, and race were recorded. We obtained data on patient’s disease characteristics, such as seropositivity, disease duration, baseline DAS28, and DMARDs, from their medical records. 

Disease activity at the time of recruitment was recorded and scored using the 28-joints-based disease activity score and using the erythrocyte sedimentation rate (DAS 28-ESR [11]. Patients were categorized as having high disease activity (DAS28 > 5.1), moderate disease activity (3.2 < DAS28 ≤ 5.1), low disease activity (2.6 < DAS28 ≤ 3.2), and being in remission (DAS28 ≤ 2.6). 

RA-related joint damage was assessed based on radiographs of the hands and feet by a musculoskeletal radiologist who was blind to the subjects’ details. The Modified Sharp score (MSS) was used to determine the severity of the radiographic joint damage [12] by a single radiologist who was blind to the subjects. The patient’s functional capacity in performing daily activities was scored using the Health Assessment Questionnaire –Disability Index (HAQ-DI) [13]. 

Based on the European League Against Rheumatism (EULAR) response criteria, patients were further divided into good, moderate, and non-responders. Patients with low disease activity or in remission and who had achieved an improvement in their DAS28 score of more than 1.2 from the baseline were considered good responders. Moderate responders were patients who achieved an improvement in their DAS28 scores of between 0.6 and 1.2 from the baseline and with moderate disease activity or an improvement of more than 1.2 in patients with high disease activity. The non-responders were subjects with an improvement of less than 0.6 in DAS28 scores from the baseline regardless of disease activity, with an improvement between 0.6 and 1.2 from the baseline with high disease activity [14].

### 2.4. IgG4 Analysis

About 3–5 mL of blood samples were taken from the subjects and processed in a centrifugal separator at 3000 rpm for 15 min. The separated sera were stored at −70 °C prior to the measurement. The serum concentration of IgG4 was tested using a commercially available enzyme-linked immunosorbent assay (ELISA). The kits (Elabscience, Houston, TX, USA, catalog number: E-EL-H6089) were pre-coated with the antibody specific to human IgG4. The sensitivity of the test was 83–97%, while the specificity was 60–85%.

### 2.5. Statistical Analysis

Data were analyzed using SPSS version 25 software (SPSS, Inc., Chicago, IL, USA) and GraphPad Prism software version 7.0 (GraphPad Software, Inc., San Diego, CA, USA). We used Spearman’s rank test to assess the correlation between IgG4 and the other continuous variables, such as DAS 28 scores, MSS scores, and HAQ-DI scores. To compare subjects with elevated IgG4 to those with non-elevated IgG4 levels, the data were analyzed using the Mann–Whitney test for continuous variables, which were expressed as the median (range), whereas Pearson’s Chi-Square test was used for categorical variables and expressed in numbers (percentages). Multivariate analysis was performed using multinomial logistic regression, with serum IgG4 levels (log10-transformed to normalize their distribution) as the independent variable. The data were expressed as the mean ± standard deviation (SD), counts (percentage), and median (range). The correlation coefficient and r-value were interpreted using the Statistic Corner by Mukaka et al. as a reference [15]. A *p*-value of less than 0.05 was considered statistically significant.

## 3. Results

### 3.1. Sociodemographic and Clinical Characteristics of the Study Population

A total of 200 subjects with RA were recruited in this study; however, 26 subjects were excluded from the final analyses as their IgG4 levels were unavailable due to either missing samples or rejected samples. Table 1 summarizes the sociodemographic and clinical characteristics of the 174 subjects. The mean age of these patients was 59.88 ± 12.56 years old. The vast majority of the patients were females (89%) with seropositive disease (82.8%). More than half (54.6%) of our study population received at least two DMARDs. Only a minority (5.7%) had high disease activity. 

### 3.2. Serum IgG4 Level and Its Correlation with Disease Parameters

**Figure 1:**
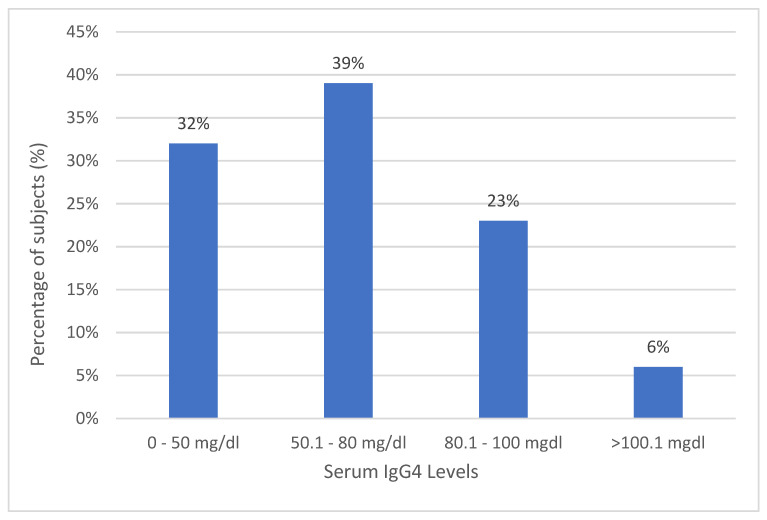
Serum IgG4 levels (mg/dL) among the RA subjects.

**Figure 2:**
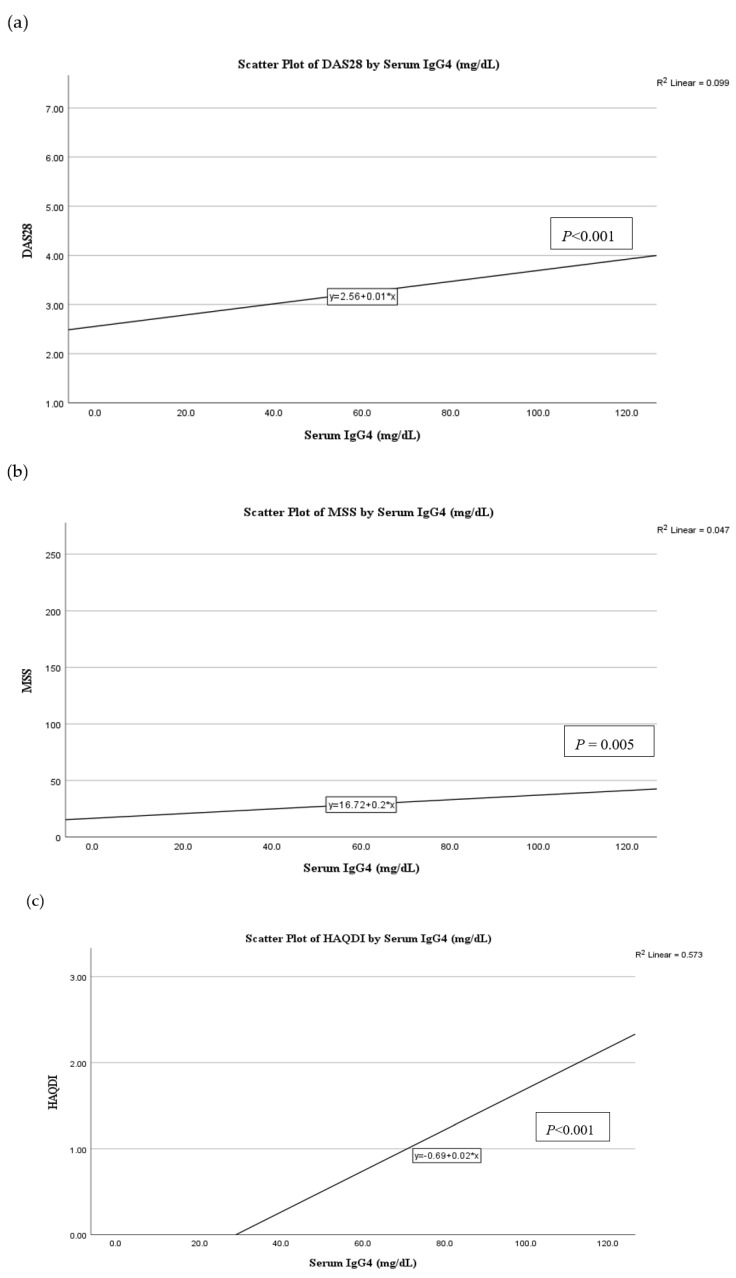
The relationship of serum IgG4 levels (mg/dL) with (**a**) The Disease Activity Score of 28 joints (DAS28), (**b**) Modified Sharp score (MSS) and (**c**) Health Assessment Questionnaire Disability Index (HAQ-DI).

The mean serum IgG4 level was 60.23 ± 30.08 mg/dL. None of the subjects had undetectable levels of IgG4 (Figure 1). Table 2 illustrates the correlation between the serum IgG4 levels and the other study parameters. Based on Spearman correlation analysis, there were significant correlations between the serum IgG4 levels and CRP (r = 0.269; *p* < 0.001), ESR (r = 0.155; *p* = 0.041), DAS28 (r = 0.406; *p* < 0.001), MSS (r = 0.195; *p* = 0.012), and HAQ-DI (r = 0.909; *p* < 0.001). Despite many variables being found to have a significant relationship with IgG4 levels, the correlations were strong (r > 0.40) for only DAS28 and HAQ-DI. Age and disease duration showed no correlations with IgG4 levels. IgG4 levels had a significant linear relationship with DAS28, MSS, and HAQ-DI (Figure 2). On multivariate correlation analysis, only HAQ-DI remained independently associated with serum IgG4 (*p* < 0.001).

### 3.3. Comparison between Subjects with Elevated and Normal Serum IgG4

The cut-off IgG4 level to diagnose IgG4-related disease was 135 mg/dL [16]. Otherwise, the definition of elevated IgG4 remained arbitrary. Based on a few studies, we used the cut-off of >86 mg/dL for elevated IgG4 [17,18]. Table 3 compares subjects with elevated and non-elevated IgG4 levels. The elevated IgG4 group had a significantly higher disease duration (*p* = 0.039), including CRP (*p* < 0.001), ESR (*p* = 0.030), DASR28 (*p* < 0.001), HAQ-DI (*p* < 0.001), and EULAR treatment response (*p* < 0.001). In terms of multivariate analysis using a multinomial logistic regression (Table 4), only HAQ-DI (OR = 4.229, 95% CI 1.302–15.751, *p* = 0.018) and DAS28(OR =3.743, 95% CI 1.062–13.193, *p* = 0.040) remained significantly associated with elevated IgG4.

## 4. Discussion

Our study demonstrated elevated serum IgG4 levels in 19.5% of the studied RA population, using a cut-off value of 86 mg/dL. Throughout the literature, the frequency of elevated IgG4 in RA has ranged widely from 6% to 46% using different cut-off levels [7,18,19,20]. The mean serum IgG4 of our studied subjects was 60.23 ± 30.08 mg/dL. The mean IgG4 levels were lower in Korean RA patients, i.e., 48.0 ± 45.4 mg/dL; however, they were much higher among the Chinese RA patients, i.e., 152 ± 27 mg/dL [7,19,21]. Variations in the mean serum IgG4 levels could be multifactorial. Nirula et al. concluded that serum IgG4 levels varied significantly even in healthy individuals with an average of 35–51 mg/dl [3].

One of the novel findings of this study was the significant positive correlation between serum IgG4 levels and RA disease activity. Disease activity in RA reflected synovial inflammation due to the upregulation of circulating cytokines such as IL-1, IL-6, and tumor necrosis factor α. The synthesis of IgG4 in vitro was regulated by IL-6. IL-6 could enhance IgG4 production via the IL-21 expressed in CD4+ T cells [22], which induces the differentiation of human naïve and memory B-cells into antibody-secreting plasma cells [23]. The association between IL-6 and IgG4 could reflect the relationship between the latter and RA disease activity. In keeping with our findings, studies have revealed the significant infiltration of IgG4-positive plasma cells in the RA synovium, which correlated with the total synovitis score, inflammatory infiltration sub-score, CD3-positive T-cells, CD20-positive B cells, and CD38-positive plasma cells [7,21]. Our findings are consistent with two other Asian studies. Kim et al. [19] and Chen et al. [24] found that serum IgG4 levels had a significant relationship with DAS28 scores and ESR levels. 

Considering multivariate analysis, HAQ-DI had an independent association with elevated IgG4 after adjusting for confounders. The functional disability was greater in the elevated IgG4 group despite comparable median MSS scores, which reflected the severity of joint damage. Functional disability in RA is multifactorial and could be the consequence of several factors, such as joint damage, secondary osteoarthritis, entrapment neuropathies, sarcopenia, and tendinopathies [25]. To date, there are no published articles that have looked into the relationship between IgG4 and functional disability. The link between circulating autoantibodies and functional disability in RA has been reported by several studies. Shidara et al. observed that an anti-cyclic citrullinated peptide (CCP) antibody is a great predictive marker for functional disability in Japanese RA patients [26]. 

We found an association between IgG4 levels and treatment response based on EULAR response criteria. Almost all subjects with elevated IgG4 were non-responders. Studies with conventional DMARDs, as well as advanced therapies, have consistently shown a marked decline in IgG4 levels with treatment [8,27,28]. Carbone et al. showed a two to three-fold decrease in IgG4 levels with tocilizumab therapy but not in IgG1 levels which is the most frequent IgG subtype [8]. Based on the study by Bos et al. [28], although all subtypes of IgG decreased with treatment, good responders based on the EULAR response criteria had the most marked reduction in IgG4 (48% reduction). Aversa et al. found that TNFα inhibitors blocked IgG4 synthesis in human B cells [29]. Moreover, IgG4 levels tended to reduce with therapy due to the disruptions in chronic stimulation by citrullinated proteins [30]. Citrullination is an inflammation-dependent process and was suppressed by DMARDs which have anti-inflammatory properties. IgG4 levels are produced by short-lived plasma cells, which are driven by citrullinated protein [31]. 

Although IgG4 levels showed a significant correlation with radiographic joint damage based on MSS scores, the strength of this relationship was negligible. This explained the comparable median MSS scores between the elevated and non-elevated IgG4 groups. The non-elevated IgG4 group had shorter disease duration and lower disease activity which could account for the above findings. In addition, the MSS assessment included the scoring of only the hands and feet. Many RA patients have radiographic changes in larger joints, such as the knees, elbows, and shoulders, which could have been missed in this study. 

We acknowledge the limitations of this study. The study was designed without including a control arm comprising non-RA subjects. This was due to budget constraints. We did not perform measurements of the total IgG. The percentage of IgG4 over the total IgG could lend credence to our findings. 

## 5. Conclusions

In conclusion, this study suggests that serum IgG4 is a potential biomarker of disease activity and functional disability in RA. Our findings may be considered preliminary and should be interpreted with caution. Further studies in this regard are much needed to consolidate our findings. 

## Figures and Tables

**Table 1:** Sociodemographic and clinical characteristics of subjects.

Parameters	N = 174
Age (years)	59.88 ± 12.56
Gender, n (%)	
Male	19 (10.9%)
Female	155 (89.1%)
Ethnicity, n (%)	
Malay	95 (54.6%)
Chinese	56 (32.2%)
Indian	23 (13.2%)
Disease Duration (years)	9.56 ± 7.82
Seropositive, n (%)	144 (82.8%)
Number of DMARDs, n (%)	
0	4 (2.3%)
1	62 (35.6%)
2	95 (54.6%)
3	13 (7.5%)
Conventional DMARDs, n (%)	
Methotrexate	109 (62.6%)
Leflunamide	58 (33.3%)
Sulfasalazine	47 (27%)
Hydroxychloroquine Advanced Therapy, n (%)	55 (31.6%)
Janus Kinase Inhibitor	14 (8.0%)
Tocilizumab	3 (1.7%)
TNF inhibitors	2 (1.2%)
Cumulative Prednisolone (mg)	5286 ± 8940
CRP (mg/dL)	1.01 ± 1.60
ESR (IU/mL)	46.88 ± 25.23
DAS28	3.22 ± 1.07
Remission, n (%)	47 (27%)
Low disease activity, n (%)	35 (20.1%)
Moderate disease activity, n (%)	82 (47.1%)
High disease activity, n (%)	10 (5.7%)
MSS	28.54 ± 27.91
HAQDI	0.73 ± 0.92
EULAR response criteria	
None, n (%)	49 (28.2%)
Moderate, n (%)	72 (41.4%)
Good, n (%)	53 (30.5%)
Serum IgG4 levels (mg/dL)	60.23 ± 30.08
0–86 mg/dL, n (%)	135 (67.5%)
>86 mg/dL, n (%)	39 (19.5%)

Data are presented as either the count (percentages) or mean ± SD. DMARDs: disease-modifying antirheumatic drugs; TNF: Tumor Necrosis Factor; CRP: C-reactive protein; ESR: erythrocyte sedimentation rate; DAS28: 28 joint-based Disease Activity Score; MSS: modified Sharp score; HAQ-DI: Health Assessment Questionnaire Disability Index; EULAR: European League Against Rheumatism.

**Table 2:** Correlation between serum IgG4 levels and other study variables.

Parameters	r-Value	*p*-Value
Age (years)	0.131	0.085
Disease duration (years)	0.076	0.318
CRP (mg/dL)	0.269	**<0.001**
ESR (IU/mL)	0.155	**0.041**
DAS28	0.406	**<0.001**
MSS	0.195	**0.012**
HAQ-DI	0.909	**<0.001**

CRP: C-reactive protein; ESR: erythrocyte sedimentation rate; DAS28: 28 joint-based Disease Activity Score; MSS: modified Sharp score; HAQ-DI: Health Assessment Questionnaire Disability Index.

**Table 3:** Comparison between subjects with elevated and non-elevated serum IgG4 levels.

	Elevated IgG4 (*n* = 39)	Non-Elevated IgG4 (*n* = 135)	*p*-Value
Age (years)	62 (38–43)	62 (28–87)	0.670 ^a^
Gender ^a^, n (%)			0.666 ^a^
Male	5 (2.9%)	14 (8.0%)	
Female	34 (19.5%)	121 (69.5%)	
Disease duration (years)	12 (1–37)	8 (1–35)	**0.039 ^a^**
Seropositive ^a^, n (%)	35 (20.1%)	109 (62.6%)	0.190 ^a^
CRP (mg/dL)	0.86 (0.07–5.80)	0.40 (0.04–12.76)	**<0.001 ^a^**
ESR (IU/mL)	52 (1–113)	43 (4–119)	**0.030 ^a^**
DAS28	3.95 (3.17–5.91)	2.91 (1–6.71)	**<0.001 ^a^**
MSS	22.5 (3–112)	22 (0–235)	0.253 ^a^
HAQ-DI	2.25 (1–3)	0.13 (0–3)	**<0.001 ^a^**
EULAR response criteria ^a^			**<0.001 ^a^**
None	38 (21.8%)	11 (6.3%)	
Moderate	1 (0.6%)	71 (40.8%)	
Good	0 (0.0%)	53 (30.5%)	

Data are presented as the median (range) or number(%) ^a^. CRP: C-reactive protein; ESR: erythrocyte sedimentation rate; DAS28: 28 joint-based Disease Activity Score; MSS: modified Sharp score; HAQ-DI: Health Assessment Questionnaire Disability Index; EULAR: European League Against Rheumatism.

**Table 4:** Multivariate analysis of disease characteristics with elevated serum IgG4 level.

Disease Characteristics	CI 95%		Odd Ratio	*p*-Value
	Lower Limit	Upper Limit		
Disease duration	0.866	1.097	0.975	0.669
CRP	0.707	2.643	1.367	0.353
ESR	0.954	1.019	0.986	0.400
DAS28	1.062	13.193	3.743	**0.040**
HAQ-DI	1.302	15.751	4.529	**0.018**

CRP: C-reactive protein; ESR: erythrocyte sedimentation rate; DAS28:28 joint-based Disease Activity Score; MSS: modified Sharp score; HAQ-DI: Health Assessment Questionnaire Disability Index.

## Data Availability

Data is unavailable due to institutional restrictions.
